# Diagnosis and treatment of tuberculosis in adults with HIV

**DOI:** 10.1097/MD.0000000000030405

**Published:** 2022-09-02

**Authors:** Qiaoli Yang, Jinjin Han, Jingjing Shen, Xinsen Peng, Lurong Zhou, Xuejing Yin

**Affiliations:** a Department of Infectious Diseases, Changzhi Medical College, Changzhi, Shanxi Province, China; b Department of Infectious Diseases, Changzhi people’s Hospital, Changzhi, Shanxi Province, China; c Department of Cardiology, Changzhi Medical College, Changzhi, Shanxi Province, China; d Department of Neurology, Changzhi Medical College, Changzhi, Shanxi Province, China.

**Keywords:** diagnosis, drug treatment, HIV, tuberculosis

## Abstract

*Mycobacterium tuberculosis*, the causative agent of tuberculosis (TB), continues to pose a major public health problem and is the leading cause of mortality in people infected with human immunodeficiency virus (HIV). HIV infection greatly increases the risk of developing TB even before CD4+ T-cell counts decrease. Co-infection provides reciprocal advantages to both pathogens and leads to acceleration of both diseases. In HIV-coinfected persons, the diagnosis and treatment of tuberculosis are particularly challenging. Intensifying integration of HIV and tuberculosis control programmes has an impact on reducing diagnostic delays, increasing early case detection, providing prompt treatment onset, and ultimately reducing transmission. In this Review, we describe our current understanding of how these two pathogens interact with each other, new sensitive rapid assays for TB, several new prevention methods, new drugs and regimens.

## 1. Introduction

Tuberculosis (TB) is the second leading cause of death from infectious diseases after coronavirus disease 2019 (COVID-19). In 2020, there were an estimated 9.9 million new cases of TB and ≈1.5 million deaths from TB worldwide.^[1]^ Human immunodeficiency virus (HIV) can increase the risk of active TB up to 26 times, which leads to atypical clinical manifestations of the latter, further complicates diagnosis and treatment, and ultimately affects its prognosis. This article mainly expounds on the pathogenesis, diagnosis, treatment, and prevention of HIV-TB co-infection.

## 2. Pathogenesis

### 2.1. Interaction between TB and HIV

*Mycobacterium tuberculosis* (MTB) enters the airway in the form of aerosols. Macrophages absorb it in the alveoli and then form granulomas after the recruitment of innate immune cells and antigen-specific lymphocytes. After the body’s immune response, TB survives in granulomas in the form of latent infection. Macrophage necrosis may be a critical stage, which not only promotes the extracellular growth of MTB but also leads to damage to the lung matrix, formation of cavities, and expulsion of large numbers of bacteria from the respiratory tract, thus promoting transmission. Nevertheless, we believe that phagocytosis of MTB by macrophages can limit its growth.

By inhibiting macrophage apoptosis, consuming CD4 + T lymphocytes affecting the functional structure of granulomas and other complex immune responses, HIV makes it difficult for the body to effectively limit the growth and dissemination of MTB, and increases significantly the risk of active pulmonary TB and its extrapulmonary transmission.^[2]^ The impact of HIV infection on the spread of TB is controversial. Generally, TB complicated with HIV results in lower sputum bacterial load, fewer pulmonary cavities, and lower infectivity of TB.^[3]^ However, for the population, HIV increases the incidence rate of active TB after exposure to MTB and may lead to the further spread of drug-resistant TB. Some researchers believe that HIV significantly affects the immune environment and may affect the selective differentiation of MTB. For example, HIV has an impact on the directional selection of the three codon sites: celAb2, katG, cyp138. Moreover, MTB could increase replication, promote activated transcription and affect diversity for HIV. The coordination and cooperation of HIV and MTB promotes each survival and development, which finally constitutes a fatal threat to patients.

### 2.2. TB-associated immune reconstitution inflammatory syndrome

TB-associated immune reconstitution inflammatory syndrome (TB-IRIS) occurs in ≈15% of HIV-infected individuals after initiation of antiretroviral therapy.^[5]^ It is a broadly immune-mediated dysregulated inflammatory response caused by activating inflammatory corpuscles. The clinical manifestations mainly include further deterioration of confirmed TB (paradoxical type) or conversion from latent pulmonary TB to active pulmonary TB (exposure type). The immune pathogenesis of TB-IRIS is complex and has not yet been fully elucidated. Studies have shown that both innate and adaptive immunity are involved in the occurrence of TB-IRIS, in which innate immunity plays a key role.^[6]^ The risk factors for TB-IRIS include when starting antiretroviral therapy, the count of CD4 + T cells is low, especially its count is < 50/mm^3^; the interval between initiation of anti-TB therapy and antiretroviral therapy is short; extrapulmonary or disseminated TB; HIV viral load is high. Currently, the diagnosis of TB-IRIS is mainly based on clinical manifestations and imaging. French scholars have found that T-cell activation markers may help predict and diagnose TB-IRIS.^[7]^ Anti-inflammatory therapy with corticosteroids is required when paradoxical TB-IRIS is refractory to treatment.^[8]^ In a clinical trial using steroids to prevent TB-IRIS, prednisone (40 mg/day for 2 weeks + 20 mg/day for 2 weeks) reduced the incidence of TB-IRIS from 47% in the placebo group to 33% in the prednisone group.^[9]^

## 3. Diagnosis

Patients with HIV-TB co-infection have low immunity and a lower bacterial load in the sputum; therefore, their clinical and imaging manifestations are more atypical. The detection rates of conventional methods for TB, such as sputum smear, sputum culture, tuberculin skin test, and interferon (IFN)-γ release test, are also low. Autopsy research in South Africa showed that up to 45.8% of HIV-infected patients fail to diagnose TB before death.^[10]^ Currently, the proportion of TB among HIV-infected individuals may be greatly underestimated. Rapid diagnostic methods for TB based on nonrespiratory specimens have been proposed to improve the diagnostic efficiency of TB in HIV-infected people.

### 3.1. Screening tests for TB in HIV-infected persons

The interferon-gamma release assay for MTB can detect IFN-γ released by specific effector T lymphocytes, which is stimulated by specific TB antigens. It is of great significance for screening latent TB infection in HIV-infected patients. A multicentre prospective study of 1510 people with HIV (PLWH) in the United States was conducted to evaluate the diagnostic characteristics of the TB skin test (TST), QuantiFERON Gold In-Tube (QFT), and T.SPOT-TB (TSPOT). This study found that TSPOT had a significantly higher positive predictive value (90.0%) than the TST (45.4%) or QFT (50.7%) and was more applicable to US-born PLWH at low risk for TB exposure and with high CD4 + counts.^[11]^

Rapid, sensitive, and low-cost screening tests for TB are even more important in areas with a high burden of HIV and TB. Among PLWH, C-reactive protein (CRP) was the first inexpensive, immediate, and effective screening test for TB (sensitivity ≥ 90% and specificity ≥ 70%) that met World Health Organization (WHO) requirements.^[12]^ It is a nonspecific marker of the acute phase of the systemic inflammatory response, which can be measured using finger-prick blood collection. Lipoarabinomannan (LAM), the lipopolysaccharide component of the cell wall of MTB, is a low-cost and point-of-care assay that can be performed in untreated urine within 25 minutes. A new generation of lateral flow lipoarabinomannan (LF-LAM) has optimized the sensitivity of LAM detection in urine. In 2019, the WHO explicitly recommended using LF-LAM in hospitalized HIV-positive patients regardless of their CD4 + T-cell count. TB-LAM positivity is also an independent risk factor for mortality in people with HIV/TB double infection.^[13,14]^ It has been incorporated into the latest guidelines of countries such as Malawi and South Africa as a screening test.

The case finding (ICF) algorithm recommended by the WHO for PLWH consists of symptom-based screening followed by confirmatory testing using Xpert MTB/rifampicin (RIF) for all individuals who screen positive.^[15]^ The algorithm can exclude TB through symptom screening, but it is difficult to apply to resource-constrained areas with a high incidence of TB because of its high cost. Yoon et al^[12]^ proposed a novel ICF algorithm based on CRP combined with TB-LAM of urine, Xpert of sputum, or sputum culture. The algorithm could increase the speed for diagnosis of TB in PLWH and reduce costs without reducing the rate of case discovery. The accuracy of the Xpert test after TB screening based on CRP is similar, but the amount of Xpert analysis required for each TB case is less than half (9–4). The addition of TB-LAM did not significantly improve diagnostic efficiency compared to the current ICF algorithm. Still, it provided a same-day diagnosis for 26% of advanced patients with HIV complicated with TB. The addition of sputum culture to TB-LAM and Xpert significantly increased the yield of ICF and identified 78% of all TB cases.

### 3.2. Diagnostic tools for TB in HIV-infected persons

The next step in TB screening is to develop accurate, rapid, and comprehensive diagnostic tools. Xpert Ultra, the next generation of Xpert MTB/RIF kits, is more sensitive than standard kits. In a multicentre study on the clinical diagnostic accuracy of Xpert Ultra and conventional Xpert, the sensitivities of Xpert Ultra and Xpert were 63% and 46%, respectively, for participants with smear-negative and culture-positive sputum. In HIV-positive participants with culture-positive sputum, Xpert Ultra also had better sensitivities than Xpert (90% and 77%, respectively), and both performed similarly in detecting RIF resistance.^[16]^ In addition, the Xpert MTB/XDR assay showed high diagnostic accuracy. The sensitivity of the Xpert MTB/XDR detection of resistance was 94% for isoniazid, 94% for fluoroquinolones, 54% for ethionamide, 73% for amikacin, 86%for kanamycin, and 61% for capreomycin. The specificity was 98% to 100% for all drugs.^[17]^

High-throughput NAAT platforms can accommodate dozens of samples simultaneously and are mainly used in reference laboratories. Some platforms are polyvalent; they can detect different pathogens simultaneously or sequentially on one platform. The integration of TB and HIV testing services is currently being achieved by developing pipelines and polyvalent platforms. Abbott’s m2000 automated platform is a polyvalent platform designed to detect HIV and TB, perform RIF/isoniazid testing for MTB, and screen for first-line drug resistance. Some studies have compared the bottom real-time MTB of the m2000 automation platform with the Xpert MTB/RIF. Among HIV-infected people, both the Abottom Real-Time MTB and the Xpert MTB/RIF have a sensitivity of > 70% and a specificity of > 90%.^[18]^

The DNA sequencing method of MTB can provide comprehensive genetic information about strains, such as drug susceptibility and drug resistance, and facilitate the selection of the most effective treatment options. Still, it is difficult to apply clinically because of insufficient understanding and technical reasons.^[19]^ WHO is building and improving a sequencing database for TB to standardize genotypes, phenotypic tests for drug sensitivity testing, and other components. Comprehensive, rapid, and straightforward DNA sequencing is routinely used to guide the management of TB patients.

## 4. Treatment

Globally, nearly 1 million HIV-infected patients are estimated to have active TB yearly. Although the anti-TB drugs for this group of patients are essentially the same as those for HIV-negative patients, there are still many problems to be solved in the combined use of anti-TB therapy and antiretroviral therapy, including the optimal timing of antiretroviral therapy, the interaction between drugs, and drug tolerance.

### 4.1. Timing of the start of antiretroviral therapy

Traditionally, given the high cost, poor compliance, and drug side effects of long-term antiretroviral therapy, many clinicians have recommended delaying the initiation of antiretroviral therapy(ART).^[20]^ With the continuous update of randomized controlled trials, the count of CD4 + T cells initiating ART has increased from an initial 350/mm^3^ to 500/mm^3^.^[21,22]^ Most international guidelines recommend starting antiretroviral therapy immediately after the diagnosis of HIV infection.^[23]^ That is, a strategy of rapid antiretroviral therapy (ie, ART should be started within 7 days) should be implemented regardless of the CD4 + T-cell count.^[24,25]^ Also, studies have shown that combination therapy with ART and anti-TB drugs is generally well tolerated. Early ART initiation allows for rapid immune recovery and reduces the risk of other opportunistic infections and all-cause mortality for people with HIV-TB co-infection. Despite the increased risk of paradoxical TB-IRIS, it is recommended that patients with CD4 + T-cell count < 50/mm^3^ and without consideration of tuberculous meningitis initiate antiretroviral therapy within 2 weeks of starting TB therapy.^[24]^ For patients with CD4 + T cell count ≥ 50/mm^3^, it is recommended to start antiretroviral therapy within 2 to 8 weeks of anti-TB therapy.^[9]^ In South Africa, waiting for TB test results is the main reason for ART treatment delays. Therefore, the SLATE II study allows LAM-negative patients with mild TB symptoms to start ART immediately without waiting for TB test results. The results suggest that early antiretroviral therapy benefits patients with TB symptoms and that rapid ART therapy are not a problem in patients diagnosed with TB after treatment.^[26]^

In primary care settings, the identification and diagnosis of TB can be slow; thus, ART initiation is delayed. It is common in primary healthcare settings to initiate empiric TB treatment without bacteriological confirmation.^[27]^ Blanc et al^[28]^ recruited HIV-infected individuals with CD4 + T-cell counts < 100 cells/mm^3^ who did not receive ART treatment in areas with a high prevalence of TB and HIV, such as Côte d’Ivoire. The patients were divided into 2 groups. The first group completed Xpert MTB/RIF, LAM, chest X-ray, and other TB screening and received an anti-TB treatment called scientifically guided treatment. The other group started empiric anti-TB treatment immediately. They found that empiric anti-TB treatment was not superior to scientifically guided treatment but had a higher probability of grade 3 or 4 adverse events.

### 4.2. New regimens and new drugs for multidrug-resistant TB

Multidrug-resistant tuberculosis (MDR-TB) arises from the mutational selection of Mtb during first-line anti-TB treatment, which leads to resistance to rifampicin and isoniazid.^[29]^ Ten percent of these patients develop resistance mutations to fluoroquinolones and second-line injectable drugs due to improper treatment, which leads to extensively drug-resistant TB (XDR-TB).^[30]^ DR-TB treatment is more complex, has a higher failure rate, and is more expensive than susceptible TB. The global treatment success rate for MDR/rifampicin-resistant TB in 2020 is 59%.^[31]^ HIV-positive patients have up to 2 to 4 times the risk of death when treated for multidrug-resistant TB. ART and high-quality anti-TB treatment can reduce mortality in HIV patients with MDR-TB.^[32]^ Improved drug regimens for drug-resistant TB are critical for controlling the acquisition and transmission of drug-resistant TB in HIV-endemic settings.^[29]^

The NIX-TB trial is a single-arm, open-label study conducted in South Africa.^[33]^ The 26-week safety and efficacy of the new regimen BPaL (a combination of bedaquiline, linezolid, and pretomanid) were evaluated in patients who had failed second-line therapy for XDR-TB or MDR-TB. The study included 109 patients, of whom 56 (51%) were HIV-positive. The study showed that 98/109 (90%) patients had good results 6 months after treatment, but the toxic side effects of linezolid were also obvious. Peripheral neuropathy and myelosuppression occurred in 81% and 48% of the patients, respectively. Following the NIX-TB trial, the ZeNix regimen completed questions regarding the dose and safety of linezolid in the BPaL regimen. This study found that by reducing the dose and duration of linezolid, toxicity could be reduced without compromising efficacy.^[34]^ Although the BPaL regimen has shown a high success rate in treating patients with XDR-TB in South Africa, its use is only conditionally recommended because of very low-quality evidence underpinning the recommendation.^[35–37]^ More controlled studies are still needed to prove whether BPaL is safer and more effective than other anti-TB regimens. Ongoing clinical trials, such as endTB and TB PRACTECAL, are expected to provide high-quality and direct evidence for short-duration and all-oral drug-resistant regimens.^[38,39]^

### 4.3. Potential drug-drug interactions

Interactions between antiretroviral and anti-TB drugs, especially those involving rifamycin, require attention. Raltegravir, an integrase strand transfer inhibitor, is widely recommended as a first-line maintenance antiretroviral therapy (ART) and efavirenz to be co-administered with rifampicin in the treatment of HIV patients with TB. However, there still remains controversy over whether it is necessary to adjust the dosage of raltegravir when combined with rifampicin. The guidelines recommend that patients taking both raltegravir and rifampicin use a double dose of raltegravir. However, Grinsztejn et al showed that in HIV patients receiving rifampicin for TB, a dose of 400 mg twice daily of raltegravir was as effective and better tolerated as the currently recommended dose of 800 mg twice daily.^[40]^ Compared with tenofovir, tenofovir alafenamide has a lower risk of side effects such as osteoporosis and nephrotoxicity and does not require dose adjustment when co-administered with rifampicin.^[41]^

Bedaquiline is a new and effective diarylquinoline(class) drug for the treatment of drug-resistant TB. Bedaquiline-containing regimens are associated with reduced mortality, quicker time to sputum culture conversion, and higher MXDR-TB treatment success, including in HIV patients. Bedaquiline is metabolized by cytochrome p450 3A (CYP3A). When co-administered, efavirenz-induced CYP3A production resulted in a decrease in bedaquiline concentration. Therefore, WHO recommends that consideration be given to changing the antiretroviral regimen from efavirenz to nevirapine when using bedaquiline.^[42]^ Delamanid is also a novel antibacterial drug used to treat patients who are resistant to first-line anti-TB drugs, and both it and Bedaquiline can prolong the QTc interval. Given the possible increased QTc-related cardiac risk, the WHO recommends not using Bedaquiline and Delamanid simultaneously. The ACTG A5343 trial compared the effects of Bedaquiline, Delamanid, and the combination of the 2 on the QTc interval in patients with MDR-TB or rifampicin-resistant TB. The combination of Bedaquiline and Delamanid had a culture conversion rate of 95% at the 8th week, showing a high early culture conversion rate, and there were no grade 3 or 4 adverse QTc prolongation events. This study provides evidence for the concomitant use of Bedaquiline and Delamanid in patients with MDR-TB or rifampicin-resistant TB (including HIV-infected patients).^[43]^

The incidence of adverse effects of anti-TB drugs, especially drug-induced liver injury, is higher in HIV-infected patients.^[44]^ For patients with HIV-TB co-infection, it is necessary to pay attention to drug interactions and monitor adverse drug reactions. If necessary, monitoring blood drug concentrations can be used to guide treatment.

### 4.4. Host-directed therapy for TB

Host-directed therapy (HDT) for TB involves various immunological, biological, and pharmacological interventions. The aim is to alter the host response to M. TB infection, which can be broadly divided into boosting the immune response to improve bacterial clearance and combating hyper inflammation to prevent tissue damage. Attention must be paid to issues related to HIV and TB co-infection.

Vitamin D is a fat-soluble vitamin with immune regulation function, which can fight against intracellular virulent MTB through the activation of macrophages. At the same time, it can inhibit the replication of HIV-1 in macrophages. However, in clinical research, vitamin D appears to be ineffective for treating TB in HIV-infected adults. HIV could block the ability of macrophages cytokine: Vitamin D3, IL-1β, IFN-γ and TNF-α both alone and in combination to response to MTB infection, which may explain this outcomes.^[45]^ A randomized controlled trial showed that among HIV-infected adults starting ART, vitamin supplementation had no effect on mortality risk and incidence of TB but may reduce the incidence of TB with positive sputum smear and high bacterial load. Further research is needed on vitamin D3 in patients with HIV/TB co-infection before implementing a treatment regimen supplemented with vitamin D3.^[46]^ Granulocyte-macrophage colony-stimulating factor (GM-CSF) plays a unique and critical role in controlling MTB infection. Exogenous supplementation with GM-CSF can activate HIV-infected macrophages to kill MTB and improve its bactericidal effect.^[45]^ Myeloid-derived suppressor cells (MDSCs) impair the proliferation, function, and trafficking of CD4 + T lymphocytes and CD8 + T lymphocytes and alter the host protective responses of people with HIV/TB co-infection.^[47]^ The therapeutic effects of drugs that inhibit MDSCs, such as tyrosine kinase inhibitors, all-trans retinoic acid, and PDL1, can be further studied in patients with HIV-TB co-infection.

New treatments are also expected to achieve good results in HIV-infected patients. Pi et al^[48]^ were the first to report the bactericidal effects of macrophage-targeted selenium nanoparticles (Se NPs) against MTB. A novel nanomaterial was proposed to assist anti-TB treatment; that is, it preferentially enters macrophages through Ison@Man–Se NPs, accumulates in lysosomes, and releases isoniazid to achieve synergistic antibacterial and bactericidal effects. This approach has broad innate immune regulatory functions and a rather low cytotoxicity and is expected to be one of the more effective treatments for drug-resistant TB.

## 5. Preventive treatment of TB

The addition of TB preventive therapy (TPT) to antiretroviral therapy has been shown to reduce TB incidence by 30% and mortality by 35% to 50%.^[25]^ The recommended regimens for treating HIV complicated with latent TB include the administration of rifapentine and isoniazid once a week for 12 weeks, rifampicin daily for 4 months, or isoniazid and rifampicin daily for 3 months. Recommended alternatives are daily treatment with isoniazid for 6 or 9 months or daily treatment with rifapentine and isoniazid for 1 month.^[24]^ Among them, daily oral rifapentine and isoniazid for 1 month were equivalent to isoniazid alone for 9 months, and the 1-month group had better compliance and fewer adverse effects.^[49]^ The study by Dooley et al^[50]^ showed that a dolutegravir-based regimen could be safely used with a weekly regimen of rifapentine and isoniazid for 12 weeks.

In addition, WHO recommends the use of isoniazid prophylactic therapy for TB (IPT) and antiretroviral therapy during pregnancy. However, because pregnant women are often excluded from IPT trials, there are relatively little data on their safety and efficacy. Gupta et al^[51]^ suggested that the immediate initiation of IPT during pregnancy may be riskier than postpartum IPT. A randomized noninferiority study showed that IPT during pregnancy increased the risk of adverse pregnancy outcomes and recommended that healthy pregnant women who had received ART start IPT at 12 weeks postpartum.^[52]^

## 6. Conclusions

In recent years, research and innovation achievements in the diagnosis, treatment, and prevention of TB worldwide have changed with each passing day. Nonetheless, TB remains one of the deadliest infectious diseases facing humanity. Under the novel coronavirus pandemic background, the number of TB cases detected and reported in 2020 has dropped sharply. The continuous global TB mortality rate decline in recent years has been interrupted. Interruption of diagnosis and treatment has seriously affected the prognosis of patients with TB. HIV infection results in higher incidence of drug-resistant tuberculosis, poor treatment effects, and increased mortality that makes a grim situation of tuberculosis control in the world. In short, we should take actions to increase investment in research, expand the coverage of ART and TPT, and delve into the genetics, immunology, and other contents of HIV-TB for developing new intervention measures against TB, such as new vaccines and immunotherapies, finally eradicate the two infectious diseases.

## Author contributions

QY, JH, JS, and LZ participated in design, methodology and manuscript edit. XP and XY participated in literature search, data collection, and manuscript editing.

Conceptualization: Lurong Zhou,Qiaoli Yang.

Methodology: Qiaoli Yang,Jinjin Han,Jingjing Shen.

Project administration: Lurong Zhou, Qiaoli Yang.

Supervision: Lurong Zhou, Qiaoli Yang,Xinsen Peng.

Validation: Lurong Zhou, Xuejing Yin.

Writing – Original Draft: Qiaoli Yang,Jinjin Han.

Writing – Review & Editing:Qiaoli Yang,Jinjin Han,Jingjing Shen,Xinsen Peng,Lurong Zhou,Xuejing Yin.
